# Translating and validating a Training Needs Assessment tool into Greek

**DOI:** 10.1186/1472-6963-7-65

**Published:** 2007-05-02

**Authors:** Adelais Markaki, Nikos Antonakis, Carolyn M Hicks, Christos Lionis

**Affiliations:** 1 Clinic of Social and Family Medicine, Dept. of Social Medicine, University of Crete, P.O. Box 2208, 71003, Heraklion, Greece; 2 Regional Health and Welfare System of Crete, Smyrnis 26, 71201, Heraklion, Greece; 3 Health Center of Anogia, 74051, Anogia, Crete, Greece; 4 School of Health Sciences, University of Birmingham, Birmingham, UK

## Abstract

**Background:**

The translation and cultural adaptation of widely accepted, psychometrically tested tools is regarded as an essential component of effective human resource management in the primary care arena. The Training Needs Assessment (TNA) is a widely used, valid instrument, designed to measure professional development needs of health care professionals, especially in primary health care. This study aims to describe the translation, adaptation and validation of the TNA questionnaire into Greek language and discuss possibilities of its use in primary care settings.

**Methods:**

A modified version of the English self-administered questionnaire consisting of 30 items was used. Internationally recommended methodology, mandating forward translation, backward translation, reconciliation and pretesting steps, was followed. Tool validation included assessing item internal consistency, using the alpha coefficient of Cronbach. Reproducibility (test – retest reliability) was measured by the kappa correlation coefficient. Criterion validity was calculated for selected parts of the questionnaire by correlating respondents' research experience with relevant research item scores. An exploratory factor analysis highlighted how the items group together, using a Varimax (oblique) rotation and subsequent Cronbach's alpha assessment.

**Results:**

The psychometric properties of the Greek version of the TNA questionnaire for nursing staff employed in primary care were good. Internal consistency of the instrument was very good, Cronbach's alpha was found to be 0.985 (p < 0.001) and Kappa coefficient for reproducibility was found to be 0.928 (p < 0.0001). Significant positive correlations were found between respondents' current performance levels on each of the research items and amount of research involvement, indicating good criterion validity in the areas tested. Factor analysis revealed seven factors with eigenvalues of > 1.0, KMO (Kaiser-Meyer-Olkin) measure of sampling adequacy = 0.680 and Bartlett's test of sphericity, p < 0.001.

**Conclusion:**

The translated and adapted Greek version is comparable with the original English instrument in terms of validity and reliability and it is suitable to assess professional development needs of nursing staff in Greek primary care settings.

## Background

Effective human resource management begins with capturing the profile and professional needs of all groups comprising the health care workforce locally, regionally or country-wide. To this end, the adaptation and use of scales and questionnaires already validated in another language proves invaluable. Cross-cultural adaptation of instruments elicits standardized data that can be used in clinical practice, teaching, research, policy making and also facilitates the exchange of information within the international scientific community [1]. In Greece, there are several studies on professional development needs of nursing personnel working in secondary and tertiary care [2,3], but studies in primary care are lacking. Although registered nurses are formally qualified to work at any level of health care provision, questions have been raised about their preparedness to adequately function in the community [4].

In line with a project initiated in 2001 by the regional health authorities of Crete to identify the training needs of nursing staff in primary health care [5], the need for a widely-accepted, practical tool for researchers and administrators became a priority. With primary care reform being at the center of a public debate in contemporary Greece, the usefulness of such a tool would be twofold. First, it would further confirm or dispute the emerging occupational profile of nursing staff from the first survey carried out in Crete having used the newly developed instrument "Assessment of Nursing Practices and Needs in Primary Health Care" [5]. Second, it would allow for information exchange with colleagues and professional organizations from other countries, especially EU members, in order to plan joint continuing education courses and professional development opportunities for nurses, midwives and health visitors.

A thorough literature search did not reveal any relevant instrument in the Greek language, whereas there were several studies using an English psychometrically valid and reliable instrument, developed by Hicks, Hennessy and Barwell [6]. The Training Needs Assessment (TNA) questionnaire has been adapted and used in several English-speaking countries and its record of use with various health care professionals is well-documented [7-9]. Recently, as part of a state commissioned study, this tool has also been successfully translated and validated in Indonesia, a heavily populated, multicultural developing country with great deficit in nursing workforce [10].

Hence, the use of a tool tested successfully both in developed English-speaking countries as well as in a large developing country would draw on the commonalities and differences experienced by Greek primary health care (PHC) professionals and offer input to the international debate. This article reports on the translation and validation of the TNA questionnaire and discusses several possibilities for implementation in the Greek primary health care setting.

## Methods

### Questionnaire

The original, self-administered, English version of the TNA questionnaire consists of four sections. Based on the developer's recommendation, a modified version was used, consisting of two sections and a biographical cover sheet with 8 short questions on demographics and research involvement. Section A consisted of 30 items within the following five superordinate categories: research/audit, communication and teamwork, administrative/technical, management/supervisory and clinical. All 30 items had to be rated along four seven-point ordinal scales. The first of these scales (A) asked respondents to assess how important the task was to their job, providing an overall occupational profile. The second scale (B) was a self-assessment of respondents' current performance level of the task, providing an index of skill. The comparison between the importance rating and the performance rating (A minus B) was the training need index, where high importance and low performance indicated a significant training need [10]. This protocol follows the importance/performance comparison approach to the assessment of training needs advocated by Martilla and James [11]. The third scale (C) evaluated the degree to which organizational changes in practice could improve performance in each task. Last, scale D measured the degree to which appropriate training would enhance performance level on each task. At the end of the questionnaire, section B comprised of an open question that gave an opportunity to the respondent to identify topics or clinical areas for further training, starting with the highest priorities.

### Translation

Based on procedures set by the Clinic of Social and Family Medicine at the University of Crete, written permission was obtained by the original developers to proceed with the translation and use of the tool for research purposes only. The Minimal Translation Criteria were followed [12] with two independent bilingual health professionals forward translating the questionnaire. One other native English speaker who did not have knowledge of the original instrument, then back translated the re-conciliated Greek version. The backward translation was sent to the developer of the original questionnaire for comparison and her suggestions were incorporated into the final Greek re-conciliated version.

Next, a cognitive debriefing process was used to identify any problems with language and to assess the degree to which a respondent's understanding of each item matched the content that was meant to elicit [12]. As part of this process, the re-conciliated Greek version of the TNA was pilot tested with 10 health care professionals who had PHC experience and were not at that time employed by any of the rural Primary Health Care Centres (PHCCs) in Crete. The pilot test group consisted of 4 Registered Nurses (RNs), 3 Health Visitors (HVs) and 3 Midwives. Written comments made by them in the Cognitive Debriefing Report were explored by the authors and were included in the final Greek version.

### Validation

#### Setting and target population

Following the pilot test, the translated, culturally adapted version of TNA was validated throughout the 14 rural Health Centres that serve the rural population of Crete. All 119 RNs, HVs, Midwives and Licensed Practical Nurses (LPNs), employed as of December 2004 and practicing at those Health Centres were eligible.

#### Participants and data collection

Validation activities were initiated in January 2005 and were completed a month later. The questionnaire was mailed to all 119 eligible subjects with each one assigned a code number known only to the first author (AM). Along with the questionnaire there was a cover letter explaining the purpose of the study, the researchers' affiliation and contact information, the voluntary nature of participation, while also clearly stating that answers would be confidential and that anonymity would be guaranteed in the final data reports. From those eligible, 55 respondents returned the completed questionnaires in the prepaid envelopes (test response rate 46.2%). Respondents represented all 14 Health Centres and all of the targeted professional groups. Four weeks later, 10 of the initial 55 respondents were randomly selected to complete the questionnaire for a second time and all of them agreed to do so (retest response rate 100%). The size of the retest sample (n = 10) was calculated as prescribed by Walter et al [13] with the following assumptions:

1) Type I error = 0.05, 2) Type II error = 0.20, 3) level of interclass correlation: *rho*_0 _= 0.6, *rho*_1 _= 0.9 (based on previous experience).

Internal consistency and reproducibility were measured as part of the reliability testing of the translated tool. Internal consistency was determined by checking the components of the questionnaire against each other, using Cronbach's alpha and requiring a minimum value of 0.70 for group and 0.90 for individual comparisons [14]. Reproducibility was measured by calculating the overall Cohen's kappa coefficient.

To determine the questionnaire's criterion validity, the same methodology as outlined by the original developers was followed [6]. Information about the respondents' research experience contained in two questions in the biographical cover sheet of the questionnaire was coded by two of the researchers along a 6-point scale (0 = no research involvement, 5 = significant involvement). These scores were then correlated with relevant research item scores from section A of the questionnaire.

To check the validity of the instrument's structure, an exploratory factor analysis using a Varimax (oblique) rotation and subsequent Cronbach's alpha was carried out on all 55 completed questionnaires, identifying separate factors that comprise the TNA questionnaire and highlighting how the items group together. A Bartlett's test of sphericity with p < 0.05 and a Kaiser-Meyer-Olkin (KMO) measure of sampling adequacy of ≥ 0.6 were used in performing this factor analysis. A factor was considered as important if its eigenvalue exceeded 1.0 [15].

#### Ethics

The study was approved by the Scientific Committee and the Board of Trustees of the Regional Health & Welfare System of Crete (protocol # 225/7/11-5-04). Participant's informed consent was indicated by an individual's willingness to complete and return the questionnaire.

## Results

### Translation

The translated tool was completed by participants without any additional external help. The TNA Cognitive Debriefing Report revealed some problems in comprehension of completion instructions. This finding explained the large number of missing items (400 out of a total of 1200 items or 33.3%) among respondents in the pilot testing. The majority of missing items (352 or 88%) were attributable to 3 HVs who all worked out of the same office and as later on admitted, influenced one another. Previous participation in research activities was significantly related to number of missing items (p < 0.0005, df = 1) since 2 out of the 4 respondents who reported having research experience were HVs.

Following a respondent's suggestion, a sentence was added to completion instructions reading, "**For your convenience, ANSWER VERTICALLY (as of each rating A, B, C, D separately) for all questions from 1 – 30." **In the demographics section some respondents reported problems understanding the actual questions. Thus, they proposed changing the phrase "job title" to "job position" and "number of years in job" as "number of years in present job". Furthermore, in regards to research exposure, they suggested changing "nature of research" to "topic of research" and the third option to the question "Has this research been published?" was changed from "near future" to "uncertain", reflecting their degree of uncertainty and lack of feedback they frequently encountered. Age and number of years in present job were found to be significantly related to having problems understanding the questions and following instructions, with the oldest and most experienced ones having the most problems (p < 0.0001, df = 1).

### Validation

The TNA questionnaire showed a very high overall internal consistency (alpha value: 0.98, 95% CI: 0.96–1.0, p < 0.001) for individual comparison. Each column also showed very good alpha values (Table 1). The overall Cohen's kappa coefficient for reproducibility (test – retest reliability) of the questionnaire was "very good" [16] (0.928, 95% CI: 0.916–0.940 p < 0.0001). Out of 120 column items (30 items × 4 columns) 89 had very good reproducibility (Cohen's kappa coefficient > 0.81), 28 had good reproducibility (0.61–0.80) and the remaining 3 had moderate reproducibility (0.41–0.60). Reproducibility results by column were also very good, as illustrated in Table 2.

**Table 1:** Internal consistency (Cronbach's alpha by column)

**Column**	**Kappa Value**	**95% Confidence Interval**	**P**
Á (Task Importance)	0.930	0.904–0.955	< 0.0001
B (Current Performance)	0.917	0.892–0.942	< 0.0001
C (Organizational change effect on performance)	0.927	0.900–0.954	< 0.0001
D (Training effect on performance)	0.938	0.914–0.962	< 0.0001

**Table 2:** Reproducibility (test-retest reliability) by column

**Column**	**Cronbach alpha**	**95% Confidence Interval**	**P**
A (Task Importance)	0.927	0.829–0.983	< 0.001
B (Current Performance)	0.932	0.841–0.984	< 0.001
C (Organizational change effect on performance)	0.939	0.850–0.988	< 0.001
D (Training effect on performance)	0.932	0.840–0.984	< 0.001

Significant positive correlations were found between respondents' current performance levels on each of the research items (Column B) and amount of research involvement with p values between 0.012 and 0.033, as illustrated in Table 3. It appears therefore, that the translated tool has significant criterion validity in the area of research activities.

**Table 3:** Criterion validity testing for column B (Current Performance)

**Item**		
3B "Critically evaluating published research"	Rp	0,355
	P	0,012
	N	49
6B "Interpreting your own research results"	Rp	0,326
	P	0,025
	N	47
7B "Applying research results to your practice"	Rp	0,358
	P	0,012
	N	48
15B "Statistically analyzing your own research data"	Rp	0,339
	P	0,021
	N	46
21B "Writing papers on your research studies"	Rp	0,375
	P	0,010
	N	46
26B "Planning a research study"	Rp	0,314
	P	0,033
	N	46

The exploratory factor analysis yielded seven factors with eigenvalues of > 1.0 (KMO measure of sampling adequacy = 0.680 and Bartlett's test of sphericity, p < 0.001). Those factors were responsible for 76.29% of variance and rotation converged in 10 iterations, presented in Table 4 (factor loadings in brackets).

**Table 4:** Factor analysis for Training Needs (Column A minus Column B)

**Item #**	**Factor 1 (accounting for 34.71**%**variance, Cronbach's α = 0.918): Communication/Patient-centered activities**
1	Developing a relationship of trust with patients (0,864)
5	Having a good relationship with colleagues (0,844)
19	Organising your time effectively (0,813)
8	Communicating with patients face-to-face (0,776)
30	Personal adaptation to health service change (0,692)
4	Appraising your performance (0,646)
10	Treating patients (0,602)
24	Assessing patients' clinical needs (0,598)
22	Undertaking health promotion activities (0,453)
17	Planning, organising individual patient care (0,417)

**Item #**	**Factor 2 (accounting for 11.40% of the variance; Cronbach's α = 0.902): Research/Audit activities**

26	Planning a research study (0,819)
21	Writing papers on your research studies (0,778)
20	Using technical equipment (0,774)
25	Collecting relevant research information (0,766)
15	Statistically analysing your own research data (0,705)
28	Gaining access to research means (time, money, info., equipment) (0,686)
12	Gaining access to literature related to your clinical work (0,544)

**Item #**	**Factor 3 (accounting for 8.70% of the variance; Cronbach's α = 0.812): Flexibility and application of knowledge**

9	Defining viable research subjects (0,830)
7	Applying research results to your practice (0,801)
11	Introducing new ideas at work (0,605)
18	Evaluating patients' psychological and social needs (0,586)
27	Working as a team member (0,572)

**Item #**	**Factor 4 (accounting for 7.17% of the variance; Cronbach's α = 0.710): Technical/Administrative procedures**

2	Doing paperwork and routine data inputting (0,884)
29	Undertaking administrative activities (0,709)
14	Making info. available to pts. and carers (0,637)
16	Showing colleagues/students how to do things (0,533)

**Item #**	**Factor 5 (accounting for 5.53% of the variance; Cronbach's α = 0.733): Relationships/Investigations**

13	Offering feedback to colleagues (0,774)
12	Gaining access to literature related to your clinical work (0,665)

**Item #**	**Factor 6 (accounting for 4.82% of the variance; Cronbach's α = 0.618): Reflective practice**

3	Critically evaluating published research (0,826)
6	Interpreting your own research results (0,559)

**Item #**	**Factor 7 (accounting for 3.95% of the variance; Cronbach's α = 0.726): Initiative**

23	Achieving your goals with limited resources (0,787)
22	Undertaking health promotion activities (0,588)

## Discussion

The lack of consensus at a national level regarding the content of training for primary health care professionals is hardly a unique problem for the Greek health care system [8,17-20]. However, in Greece this problem is further compounded by a heavily medical-oriented culture that permeates health science curricula and shapes public expectations about primary health care services and each discipline's role in the community arena. The importance of taking into account specific local needs, along with the growing demand for continuous updating of professional knowledge and skills have resulted in a proliferation of courses that do not always meet the training needs of those being aimed at [8,17,19]. To overcome this problem, it is essential that PHC professional training must be based on a scientifically constructed needs analysis tool that could provide a systematic framework for addressing the identified needs. This systematic framework could be a response to the increasing demand for cross-cultural comparisons in the international setting and the use of common instruments and definitions valid to each culture [21].

Within the context of a Health Care Reform Act seeking to improve quality in primary care [22] and integrate health care services nationally [23], the authors identified and translated a culturally compatible instrument that would detect professional needs among various health professional groups and across the continuum of care settings. The validation process revealed a substantial Cohen's kappa coefficient and a satisfactory Cronbach's alpha, suggesting that the instrument is reliable for the Greek setting. Criterion validity was also good indicating that the instrument was valid, while the exploratory factor analysis of training needs revealed the shared variance of 7 separate factors.

However, some concerns have been raised when discussing the findings of the TNA's validation into Greek language and particularly:

a) the small targeted population and sample size, attributed to the main study's aim and subsequent design. As an offset to this inherent limitation, participants were representative of all four targeted professional groups as well as the fourteen Health Centres that serve rural Crete.

b) the relatively low return rate of 46.2%, which could be explained by having a self-selected sample, based on the cover letter's indication that the questionnaire aimed to identify training needs. Thus, similarly to past experiences [8], nursing staff who did not consider themselves in need of further training were implicitly excluded.

c) the relatively small sample size for factor analysis, which can be considered as adequate. However, there is no consensus among researchers, while there is some evidence that our sample size was adequate according to one criterion (N of cases > N of items; 55 respondents > 30 items) [24-26].

d) the missing data attributed to the lack of experience of the targeted population with research tools requiring rating of each item according to four criteria. In contrast to previous international validation studies of the TNA tool, where it had been administered in its simpler format omitting two of the four columns, our validation study included all four columns, making it more complex and demanding for participants to complete.

e) section B of the questionnaire with its open-ended structure, clearly does not have the same psychometric properties, but can be used in conjunction with section A to confirm responses regarding the participant's training needs.

Implications of the translated and validated questionnaire can be demonstrated at a local, national and international level. Locally, it is expected to provide a "hands-on" approach to front-line regional managers in detecting and documenting professional skills and needs. At a national level, health authorities will have at their disposal a valuable tool that provides baseline measurement as well as continuous monitoring of the quality and management of primary care nursing across Greece. Although validation was carried out exclusively among nursing staff in primary health care settings, there are strong indications from previous studies that it could also be psychometrically robust when used in secondary and tertiary care settings or for other health professional groups. Consequently, the newly formed Hellenic Regulatory Body of Nurses might be interested in carrying out further research in other settings, establishing new practice standards and developing policies. Furthermore, academic institutions interested in developing new programs with an emphasis on interdisciplinary primary health care could benefit from the tool's use.

At an international level, initiatives for improving primary health care nursing and strengthening team work on a larger scale than that of the Crete project have been reported [27,28]. This study aims to add one more piece to the regulatory system framework that would facilitate the international transfer of health care professionals, especially within the EU. Hence, despite its limitations, this validation study may be useful for health planners, managers, clinicians and researchers from EU countries experiencing the same conditions as Greece and attempting to implement similar quality improvement programs in primary health care.

## Conclusion

The translated questionnaire into Greek language appears to be a reliable and valid tool for identifying professional development needs of nurses, midwives and health visitors in primary health care settings. The tool has a considerable degree of flexibility, making it suitable for a variety of applications in several settings. Its feasibility as a large-scale survey instrument for use among various Greek health care professional groups in secondary and tertiary care settings remains to be tested.

## Abbreviations

PHC: Primary Health Care

TNA: Training Needs Assessment

PHCCs: Primary Health Care Centres

RN: Registered Nurse

HV: Health Visitor

LPN: Licensed Practical Nurse

EU: European Union

## Competing interests

The author(s) declare that they have no competing interests.

## Authors' contributions

All authors read and approved the final manuscript. AM participated in study design, translation and adaptation of the questionnaire, carried out data collection and data entry, participated in the analysis and wrote the final draft of the manuscript. NA participated in study design, carried out the statistical analysis and co-wrote the final draft of the manuscript. CH provided initial approval to use the instrument, consultation during translation/adaptation/validation process and co-wrote the final draft of the manuscript. CL conceived the study design, participated in the translation/adaptation/validation process and co-wrote the final draft of the manuscript.

## Pre-publication history

The pre-publication history for this paper can be accessed here:


